# Uni- versus bilateral antegrade cerebral perfusion during repair of acute aortic dissection: Still a discussed matter!

**DOI:** 10.1016/j.xjtc.2022.10.012

**Published:** 2022-10-30

**Authors:** Thierry Carrel, Martin Schmiady, Ahmed Ouda, Paul Robert Vogt

**Affiliations:** Department of Cardiac Surgery, University Hospital, Zürich, Switzerland

**Figure:**
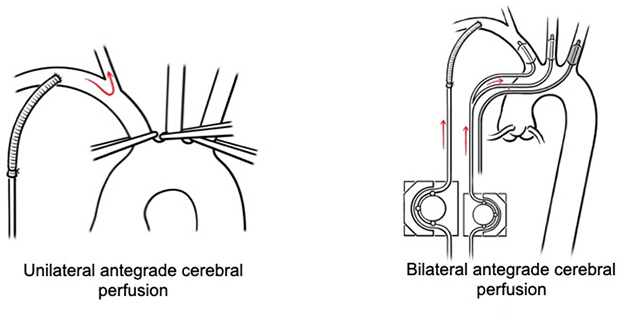
Unilateral (*left*) versus bilateral (*right*) antegrade cerebral perfusion.

**Central Message**

Cerebral perfusion during repair of acute aortic dissection is a controversial topic: attention should not only be drawn to cerebral protection but also to the manipulation of supra-aortic vessels.

Since the introduction of cerebral perfusion as a brain-protection method during the surgical repair of the aortic arch, a significant number of papers have analyzed which type of perfusion may provide the most optimal cerebral protection:

• antegrade cerebral perfusion (ACP) via the supra-aortic branches or retrograde cerebral perfusion via the superior vena cava;
• unilateral ACP performed through the subclavian artery cannula of the cardiopulmonary bypass circuit; or
• bilateral ACP with 2 selective perfusion catheters introduced in the innominate artery (respectively advanced into the right common carotid artery) and the left common carotid artery.

This topic is still a matter of debate, and highly contradictory opinions are reported in the literature. Although some surgeons defend unilateral ACP as the simplest and most efficient method of cerebral protection, other prefer to “mimic” the normal physiology and use bilateral ACP with 2 catheters combined or not to the occlusion of the left subclavian artery.^1^, ^2^, ^3^, ^4^, ^5^, ^6^ Other groups still favor retrograde cerebral perfusion and/or demonstrated that the latter method may be associated with fewer radiographic neurologic injuries than ACP or no significant clinical difference.^7^^,^^8^

In this short paper, we would like to focus on cerebral protection during repair of acute type A aortic dissection because the handling of the supra-aortic branches (clamping, snaring, occlusion with balloons) in the setting of a weakened arterial wall may cause injuries that need particular attention. Furthermore, we report on some clinical observations made following clamping of the innominate artery.

## A Controversial Discussion

In a recent paper from Vienna, 184 patients received aortic repair because of acute type A aortic dissection using bilateral (n = 91) and unilateral (n = 93) ACP. Overall, clinical outcomes were similar.^1^ However, subgroup analyses suggested that bilateral ACP was associated with superior survival in patients requiring a duration of circulatory arrest of 50 minutes or longer.

**Figure 1:**
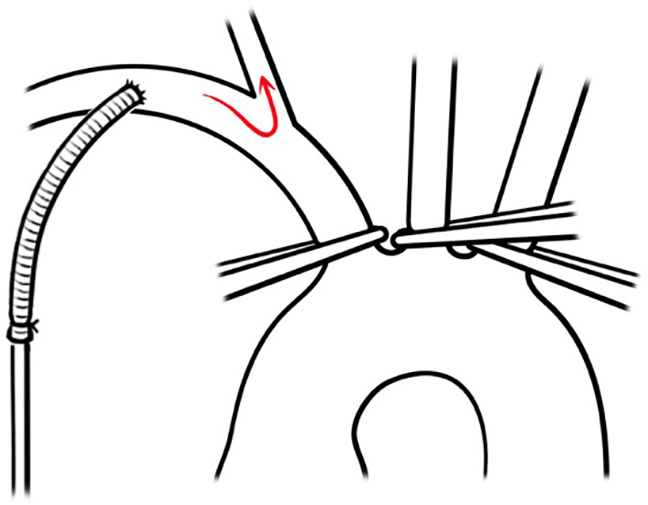
Unilateral ACP during circulatory arrest through the arterial cannula of the cardiopulmonary bypass circuit. In this case, the innominate artery must be occluded (either by a clamp or by snare with tourniquet). In addition, occlusion of the left common carotid and subclavian arteries preclude backflow through the circle of Willisi and contribute to an increased intracerebral perfusion pressure.

The decision to proceed with unilateral or bilateral ACP depended on the surgeon's preference and experience, as well as the estimation of the required duration of circulatory arrest. Before starting unilateral ACP, the surgeons at Vienna University Hospital clamped all 3 vessels (Figure 1). During ACP, oxygen saturation was monitored bilaterally using near-infrared spectroscopy. If the latter decreased by 15% to 20% during unilateral perfusion, the left common carotid artery was also cannulated and cerebral protection switched to bilateral ACP.

In this series, the rate of bilateral cerebral lesions and unilateral left-sided lesions was greater in patients receiving unilateral ACP, whereas the rate of unilateral right-sided lesions was greater in patients receiving bilateral ACP. From these findings, the authors concluded that the insertion of an additional perfusion cannula into the left common carotid artery does not increase the risk of left-sided cerebral lesions.

In contrast to this publication, Piperata and colleagues^2^ published recently the results of a retrospective, multicenter study that compared unilateral (39% of the patients) versus bilateral (61%) ACP during the repair of acute type A aortic dissection. In this study, the flow for ACP was accomplished through the right subclavian artery arterial line from the main pump of the cardiopulmonary bypass circuit (1/3) and through a separate pump for the left carotid and left subclavian arteries (2/3). The relative blood flow through these 2 separated perfusion lines was regulated according to the observed near-infrared spectroscopy values, bilateral radial artery pressure monitoring, and the pump line pressure. Using a propensity score analysis, the authors demonstrated that patients who received bilateral ACP had a significantly greater incidence of permanent neurologic deficits (*P* < .001), left brain hemisphere stroke (*P* = .007), and all-combined complications (*P* < .001). The hypothesis for this observation was that more manipulations around and within both carotid arteries during bilateral perfusion as well as some dysfunction of the cerebral autoregulation with significant unequal blood flow into both hemispheres may precipitate adverse cerebral outcomes.

## It Is Not a Matter of Perfusion Only: Handling the Supra-Aortic Branches Is Important!

**Figure 2:**
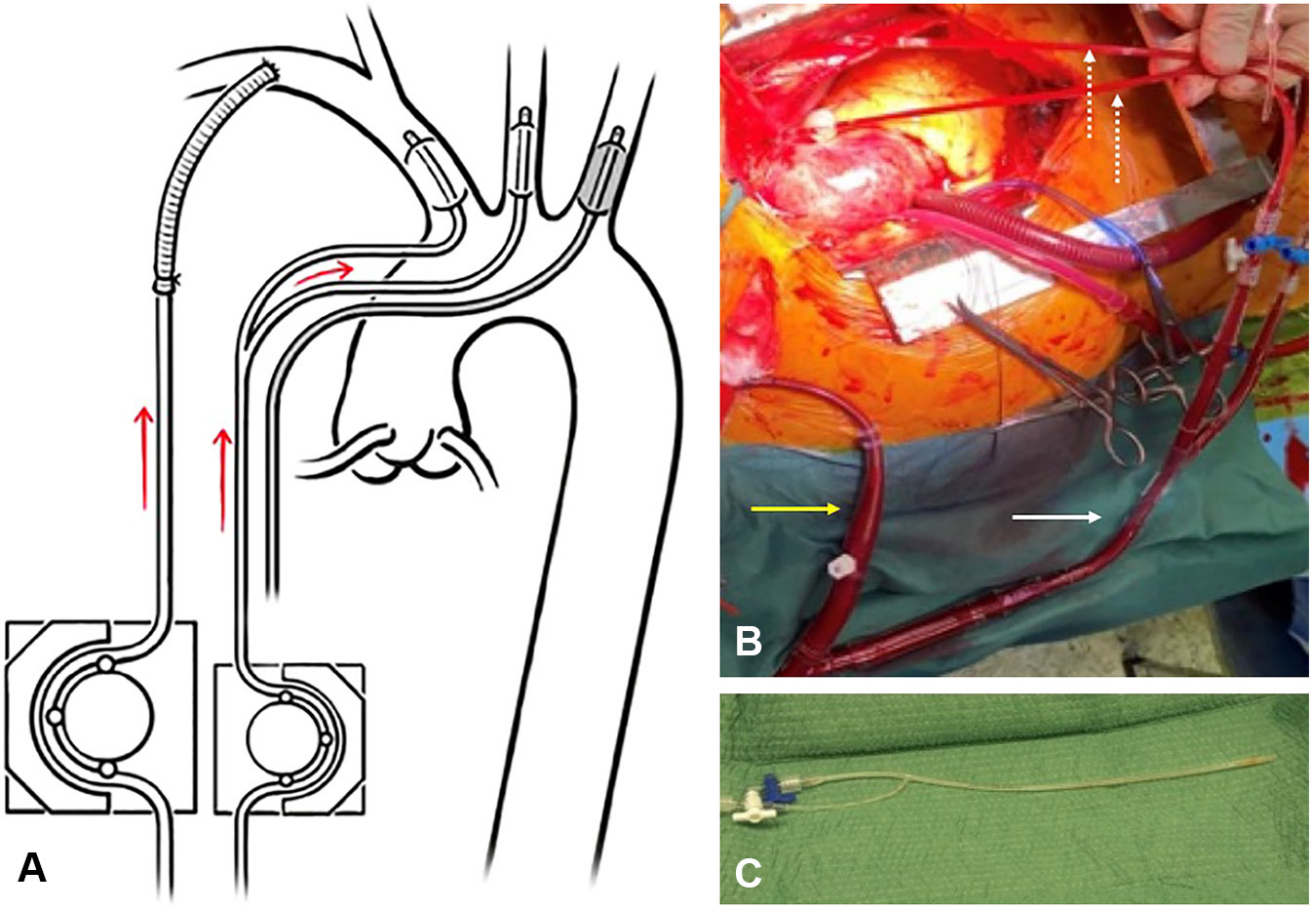
A, Bilateral selective ACP with balloon catheters. Ideally, ACP is performed through a separate line connected to a smaller cardioplegia pump. B, Alternatively, ACP may be performed through the normal arterial return line using a Y-connector and clamping the right subclavian line (*yellow arrow*: subclavian artery line; *white arrow*: additional line for the bilateral cerebral perfusion; *dotted arrows*: perfusion catheters with balloon occlusion). C, Perfusion catheter with balloon occlusion through a separate line for selective ACP.

In addition to the discussion regarding perfusion and its characteristics, we would like to add one important point, namely the concern regarding manipulation of the supra-aortic branches, especially in the setting of a weakened arterial wall. The first author of this paper introduced hypothermic circulatory arrest combined with bilateral ACP during the repair of aortic dissection and any type of aortic arch aneurysm at a previous institution in 2002 with a case load of 80 to 100 hypothermia cases per year (approximately 50% being cases of acute type A aortic dissection). Unilateral ACP was used only exceptionally, mainly when the introduction of a perfusion catheter into one of the common carotid arteries was technically not possible (kinking, severe ostial stenosis, or occlusion). The classical setting for bilateral selective ACP was an additional small pump in the cardiopulmonary bypass circuit (identical to that one used for cardioplegia) and a tubing system connected to an “octopus” with 2 arms (similar to what is used for selective cardioplegia) (Figure 2, *A*). As alternative, the ACP line is connected with a Y to the arterial return line that is clamped during ACP (Figure 2, *B*).

For the purpose of bilateral ACP, a special very smooth and small perfusion catheter with balloon occlusion was developed in the department and later commercialized by LeMaitre Vascular (Figure 2, *C*). There are 3 potential advantages to using this approach. First, there is no need to clamp or snare any of the supra-aortic vessel in the context of a particularly fragile vessel wall through acute dissection (especially when it is dissected) with the risk of an additional injury to these vessels with subsequent flow obstruction or complete occlusion. Second, the introduction of a smooth balloon into the true lumen may favor expansion of the dissected cylinder from inside the vessel and bring the dissected layers somewhat together, especially when biologic glue has been introduced in-between the layers. In fact, the balloon is inflated very gently just to avoid retrograde flow. Third, balloon inflation into the carotid arteries (or simply into the innominate artery on the right side) may obviate unperceived glue entrapment, a critical event that has been reported, when glue was routinely introduced for aortic dissection repair.^9^

## Injury During Clamping and Consecutive Occlusion of the Innominate Artery

The first author moved recently to another tertiary care center, with an annual volume of 60+ acute type A aortic dissections (2021: 82 cases) with a strategy of unilateral ACP. In a consecutive series of 55 patients operated during an 8-month interval, 5 cases (10%) of postoperative occlusion of the innominate artery were observed. All patients underwent replacement of ascending aorta with an open distal anastomosis at the level of the proximal aortic arch. ACP was performed according to the following characteristics: core temperature 26 to 30 °C, tympanic temperature <24 °C, cerebral flow 10 to 15 mL/kg body weight, and pressure 50 to 60 mm Hg. Occlusion of the innominate artery was thought to be caused or aggravated by clamping or snaring this vessel during unilateral ACP through the right subclavian arterial line. In 4 patients, the innominate artery was already dissected at preoperative computed tomography scan, and in 1 case, it was intact.

**Figure 3:**
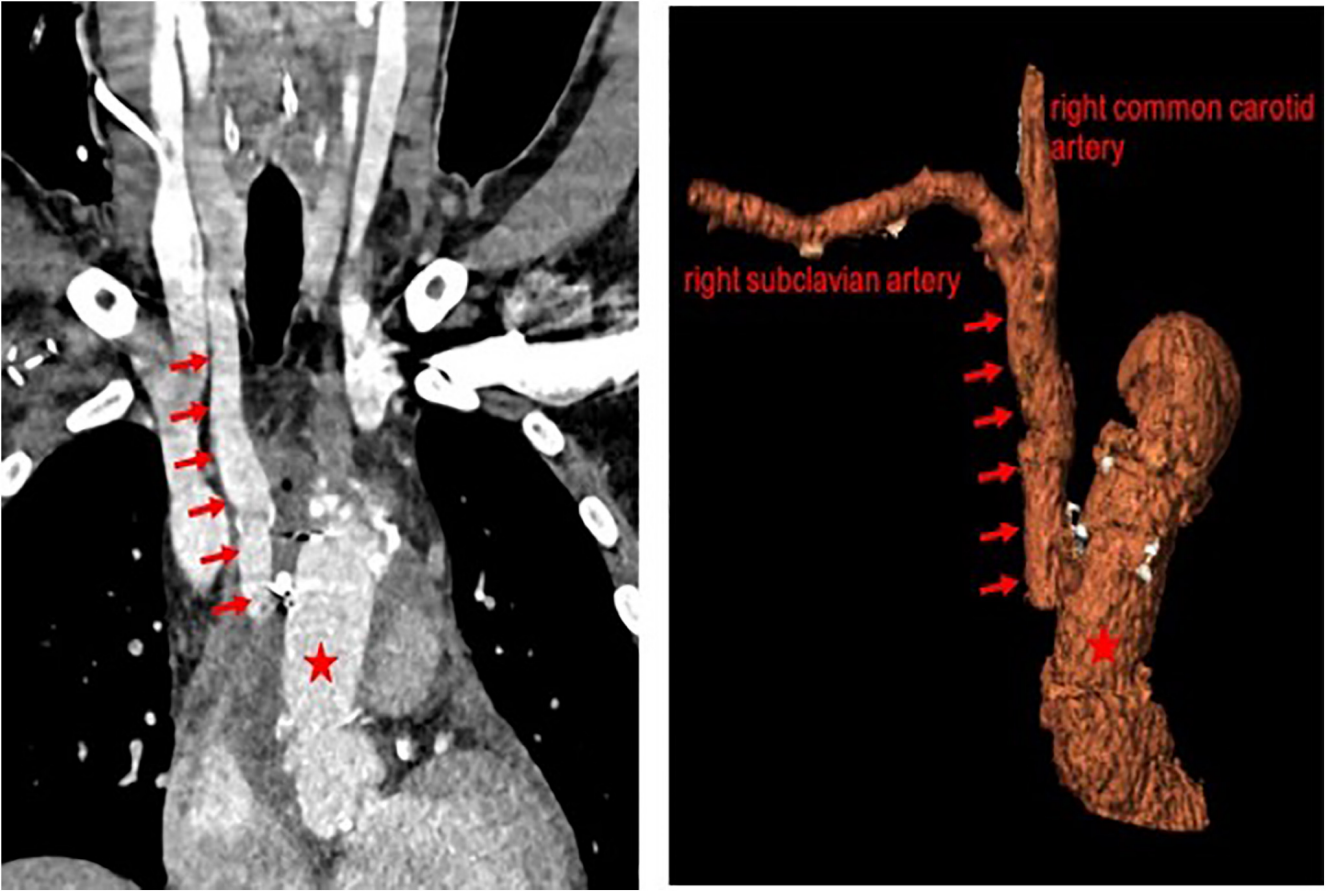
Postoperative computed tomography imaging and 3-dimensional reconstruction showing revascularization of the proximally occluded innominate artery with a vascular graft (*arrows*) between the ascending aorta (*star*) and the bifurcation of the innominate artery.

**Figure 4:**
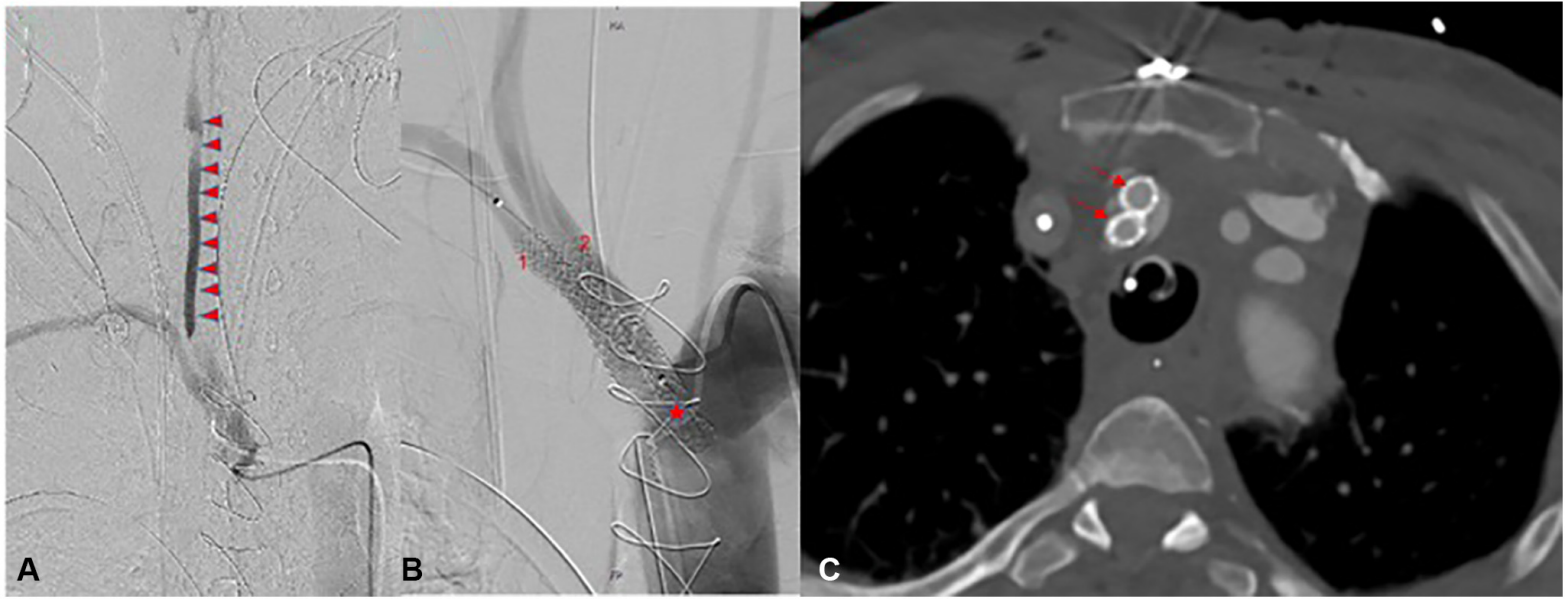
A, Angiographic imaging of the innominate artery shows retention of the contrast agent in the false lumen of the dissected common carotid artery (*arrowheads*). B, The dissected innominate artery and the right common carotid artery were treated with 2 kissing stents, which reached out into the aortic arch (*star*). The technique of the kissing stents ensures optimal distal perfusion of both the right subclavian and right carotid arteries and precludes compression or occlusion of one vessel through the other one. In addition, kissing stents allowed a safe closure of the supposed entry in the trunk, whereas vascular access in the true lumen was secured using ultrasound-guided puncture of the right carotid artery. C, Postinterventional computed angiography showing the 2 parallel stents in the innominate artery (*arrows*).

All patients suffered from a major ipsilateral neurologic injury, and 1 patient died. Two underwent emergency revascularization, with a vascular graft interposition between the ascending aorta and the bifurcation of the innominate artery in one (Figure 3) and complex stenting of the innominate artery up to the bifurcation of the right common carotid artery in the second case (Figure 4). In the 2 other cases, no additional revascularization was performed, since the dissecting membrane extended distally to the carotid artery bifurcation up to the intracranial part of the internal carotid artery, and the neurologic deficit was thought to be irreversible.

Patients who received revascularization showed significant improvement of their neurologic deficit and were discharged with a mild residual hemiparesis, whereas the 2 patients treated conservatively still suffered from significant hemiparesis at discharge to neurorehabilitation.

The institutional review board/ethical review board of our university hospital granted a waiver for this work, since all patients signed the general consent form that allows the use of anonymized data for research purposes.

## Comment

When weighing the risks and benefits of unilateral versus bilateral ACP, interest should not only focus on the quality of cerebral protection but also the potential dangers during manipulation of the supra-aortic branches, which has emerged to be extremely important as well. First, those who do not use or support bilateral ACP argue the potential danger of manipulating supra-aortic vessels during the introduction of perfusion catheters into the innominate artery and the left common carotid artery and warn about the risk of carotid dissection or embolization of atherosclerotic particles. In fact, the presence of aortic arch and branch atherosclerosis is extremely uncommon in patients presenting with acute aortic dissection. In addition, it does not seem logical to believe that potential mobilization of thrombotic material from the false lumen may be better avoided by clamping a vessel than by occluding it with an intraluminal balloon.

Second, one should be aware that clamping an already-dissected supra-aortic vessel through a vascular clamp or a tourniquet for snaring represents an additional severe trauma, which has practically never been addressed in the literature. Third, a gently expanded intravascular balloon may safely occlude the vessel and also prevent the embolization of glue or thrombotic material during the sealing process at the level of the distal aortic anastomosis when the aortic arch is open. This is also a rarely discussed potential advantage.

We have used the technique with small perfusion catheters and balloon blockage extensively in the setting of thoracoabdominal and abdominal aneurysms repair to perfuse and/or occlude either spinal or visceral arteries and to block retrograde blood flow. We never observed any compression injury, because the balloon is inflated smoothly, just until there is no backflow out of the perfused artery.

Finally, monitoring of the right radial artery pressure to assess the efficacy and safety of ACP may not be optimal because the individual positioning of the cannula in the subclavian artery and/or the angle of the side-graft anastomosed to the subclavian artery may cause significant variations in flow and pressure during perfusion.

## Conclusions

These are a few considerations regarding unilateral and bilateral ACP that should provide additional information regarding the potential dangers of clamping the supra-aortic vessels, in particular, the innominate artery when it is dissected. Endovascular balloon occlusion seems at least safer in terms of additional trauma to an already-fragile arterial wall. Nevertheless, only prospective randomized studies would be able to definitively clarify a question that surfaced in the introduction of cerebral protection more than 2 decades ago.

### Conflict of Interest Statement

The authors reported no conflicts of interest.

The *Journal* policy requires editors and reviewers to disclose conflicts of interest and to decline handling or reviewing manuscripts for which they may have a conflict of interest. The editors and reviewers of this article have no conflicts of interest.
